# Editorial: Methods and application in integrative and regenerative pharmacology: 2021

**DOI:** 10.3389/fphar.2022.1077352

**Published:** 2022-11-25

**Authors:** Peter Natesan Pushparaj, Gauthaman Kalamegam, Seeram Ramakrishna

**Affiliations:** ^1^ Center of Excellence in Genomic Medicine Research, King Abdulaziz University, Jeddah, Saudi Arabia; ^2^ Department of Medical Laboratory Technology, Faculty of Applied Medical Sciences, King Abdulaziz University, Jeddah, Saudi Arabia; ^3^ Center for Transdisciplinary Research, Department of Pharmacology, Saveetha Dental College and Hospitals, Saveetha Institute of Medical and Technical Sciences, Chennai, India; ^4^ Pharmaceutical Division, Nibblen Life Sciences, Chennai, India; ^5^ Center for Nanofibers & Nanotechnology, Department of Mechanical Engineering, National University of Singapore, Singapore, Singapore

**Keywords:** integrative and regenerative pharmacology, MiRNA-21, leptin, wound healing, intervertebral disc degeneration, biomaterials, stem cell therapy, pharmacotherapy

## Introduction

The last decade has seen notable developments in the research environment of integrative and regenerative pharmacology (Richardson et al., 2016; Huwaikem et al., 2021; Irfan et al., 2022; Rani Raju, et al., 2022). Therefore, the Integrative and Regenerative Pharmacology special issue series aims to highlight the state-of-the-art experimental methods to explore fundamental issues and application of biomolecules in stem cell biology and the use of pharmacological agents in conjunction with biotechnological and nanotechnological principles. With our research theme, we aimed to attract manuscripts that focus on recent advances in basic and translational research and expand current knowledge of integrative and regenerative methods and their applications (https://www.frontiersin.org/research-topics/28076/methods-and-application-in-integrative-and-regenerative-pharmacology-2021). Fifteen manuscripts were submitted by authors from around the world, 4 of which were accepted for publication after rigorous peer review. The remaining 11 manuscripts were not accepted for publication. In this editorial article, we discuss the research articles and reviews published on our Research Topic, focusing on the role of microRNA (miR21)) and leptin in wound healing, research trends, and hotspots in stem cell therapy for intervertebral disc degeneration (IDD) based on bibliometrics, and pharmacotherapy in experimental spinal cord injury (SCI) in rats.

## Role of miR21 and leptin in wound healing

In the Research Topic, Xie et al. reviewed the role of miR21 in wound healing. They reasoned that miR21 targets multiple biological processes and signaling pathways, especially the angiogenic and inflammatory pathways that are crucial in regulating the process of wound healing. More specifically, miR21 regulates intracellular signaling molecules such as programmed cell death protein 4 (PDCD4), phosphatase and tensin homolog (PTEN), reversion-inducing cysteine-rich protein with Kazal motifs (RECK), sprouty RTK signaling antagonist 1 (SPRY1), SPRY2, mitogen-activated protein kinase/extracellular-signal-regulated kinase (MAPK/ERK) and phosphatidylinositol 3 kinase/protein Kinase B (AKT) (PI3K/AKT) (Xie and Weiskirchen, 2020) to either promote or delay the wound healing process. The authors emphasized that the progress in the development of miRNA nanocarrier systems might improve the clinical efficiency of such miRNA-based therapy. Although the effects of miR21 on wound healing have been preliminarily elucidated, the functions of miR21 in various types of wound healing need further investigation. Nevertheless, the comprehensive review by Xie et al. helps to underpin the fact that miR21 may serve as a potential therapeutic target for wound healing.

In the comprehensive review, Yuan et al. discussed the relationship between leptin, an anti-obesity hormone, and wound healing based on studies using both *in vitro* and *in vivo* models. Based on articles published in the field of wound healing, the authors state that regulation of inflammation by immune cells, re-epithelialization, proliferation, angiogenesis, and fibroblast differentiation are essential determinants in wound healing. The authors emphasized that the wound healing process promoted by leptin involves many signalling pathways, such as activation of signal transducer and activator of transcription 1 (STAT1) and STAT3 *via* p38 MAPK or janus kinase 2 (JAK2). Importantly, the authors discussed the delayed wound healing in leptin-deficient mice and its reversal after leptin administration. However, the authors have rightly noted that although leptin promotes re-epithelialization and supports wound healing, the roles of leptin in the inflammation, proliferation, and maturation phases of wound healing are still nascent and require further research.

### Role of stem cells in intervertebral disc degeneration

Wang et al. applied systematic bibliometric and visual analysis methods to determine the role of stem cells (SCs) in intervertebral disc degeneration (IDD). The authors have comprehensively searched the Web of Science Core Collection (WOSCC), Scopus, PubMed, and ClinicalTrials.gov databases to collect the relevant literature for bibliometric and visual analysis. They used scientific software such as VOS viewer 1.6.17, CiteSpace 5.8.R.1, and Scimago Graphica for bibliometrics. Wang et al. found that most publications came from China and most international collaborations and citations came from the United States of America (USA). They identified the authors with the highest productivity and highest quality articles, the most frequently published and cited journals, and current hotspots such as mechanistic studies in SCs and IDD research. They also found studies supporting the use of tissue-engineered scaffolds of SCs in degenerative disc disease. Importantly, they found that clinical trials were rare and direct injection of mesenchymal stem cells (MSCs) into degenerated discs for the treatment of degenerative disc disease (DDD) was the latest trend in this research area. In fact, direct injection of MSCs into degenerative discs have short-term benefits which are mainly attributed to their paracrine effects rather than integration of SCs and tissue regeneration. As rightly mentioned in the article, there is a dearth of clinical trial studies and this needs to be stepped up to have more conclusive evidence to understand the role of SCs in IDD and other skeletal disorders.

### Pharmacotherapy for spinal cord injury

Hashemizadeh et al. demonstrated the benefits of early administration of bumetanide, a diuretic, in a rat model with spinal cord injury (SCI). The authors found that the chloride co-transporter Na + -K + - Cl- co-transporter isoform 1 (NKCC1) was upregulated in the center of the injured spinal cord 3 hours after lesion onset and peaked approximately 6 h after SCI. Treatment with bumetanide, an NKCC1 inhibitor, improved locomotor function and increased levels of the protein Growth Associated Protein-43 (GAP-43) 4 weeks after induction of SCI. The authors confirmed the beneficial effects of bumetanide through histologic tissue studies that exhibited neuroprotective and regenerative effects. Here, the authors provided new evidence for the beneficial effects of bumetanide when administered immediately after SCI.

## Discussion

miRNAs are endogenous short RNA molecules consisting of 20–22 nucleotides (Pushparaj et al., 2008a; Pushparaj et al., 2008b). miRNAs regulate gene expression by binding to the 3′-untranslated region (UTR) of the target gene, promoting degradation or, in some cases, activation (Li et al., 2006; Li, 2017; Tan et al., 2021). miRNAs play an important role in the regulation of various biological processes such as cell differentiation, proliferation, apoptosis, cell migration, etc. Thus, the dysregulation of miRNAs has been frequently observed in many pathologies, including wound healing and they have been proposed as therapeutic targets for several diseases.

Efficient wound healing is pivotal, and it is a chief burden for healthcare providers around the globe. Here, Xie et al. discussed the key role of miR21 in wound healing. However, based on some wound healing studies, the upregulation of miR21 decreased leptin levels in vascular ulcers (VUs) and the silencing of miR21 increased leptin levels (Pastar et al., 2012). On the other hand, similar to Xie et al. Yuan et al. pointed out that overexpression of miR21 enhanced wound healing in aged mice and that miR21 may play different roles depending on the type of wound (Long et al., 2018). Therefore, identifying and validating the biological mechanisms of miR21 and leptin in wound healing is essential for developing a therapy for wound healing.

Bibliometric studies are essential to deciphering global research trends and hotspots in a particular research area around the globe (Agarwal et al., 2021; Baskaran et al., 2021). The bibliometric study by Wang et al. revealed the global research hotspots, trends, and clinical use of SCs in the treatment of IDD. Bibliometrics helps researchers quickly grasp the hotspots and latest trends in research. Therefore, researchers can use the bibliometric methods as demonstrated by Wang et al. to decipher the necessary clues from the literature in the field of integrative and regenerative pharmacology to design and develop their research.

Disturbances of ionic balance are common in SCI because the activity of γ-aminobutyric acid (GABA) receptors depends on intracellular chloride levels. Chloride homeostasis is controlled by two co-transporters, NKCC1 and K + -Cl- co-transporter (KCC2), which are essential for chloride homeostasis. NKCC1 has been found to be disrupted in SCI (Nasirinezhad et al., 2021). The axonal pathways that control sensory and motor functions are affected in SCI, leading to neuronal impairments (O’Shea et al., 2017). Hashemizadeh et al. discovered bumetanide, an inhibitor of NKCC1, in a rat model of SCI and noted the beneficial effects of this diuretic drug. Previously, bumetanide was found to have analgesic effects on neuropathic pain in patients with SCI (Zarepour et al., 2020). Therefore, the development of new or repurposed drugs in regenerative medicine is essential for better patient care in clinics.

**FIGURE 1:**
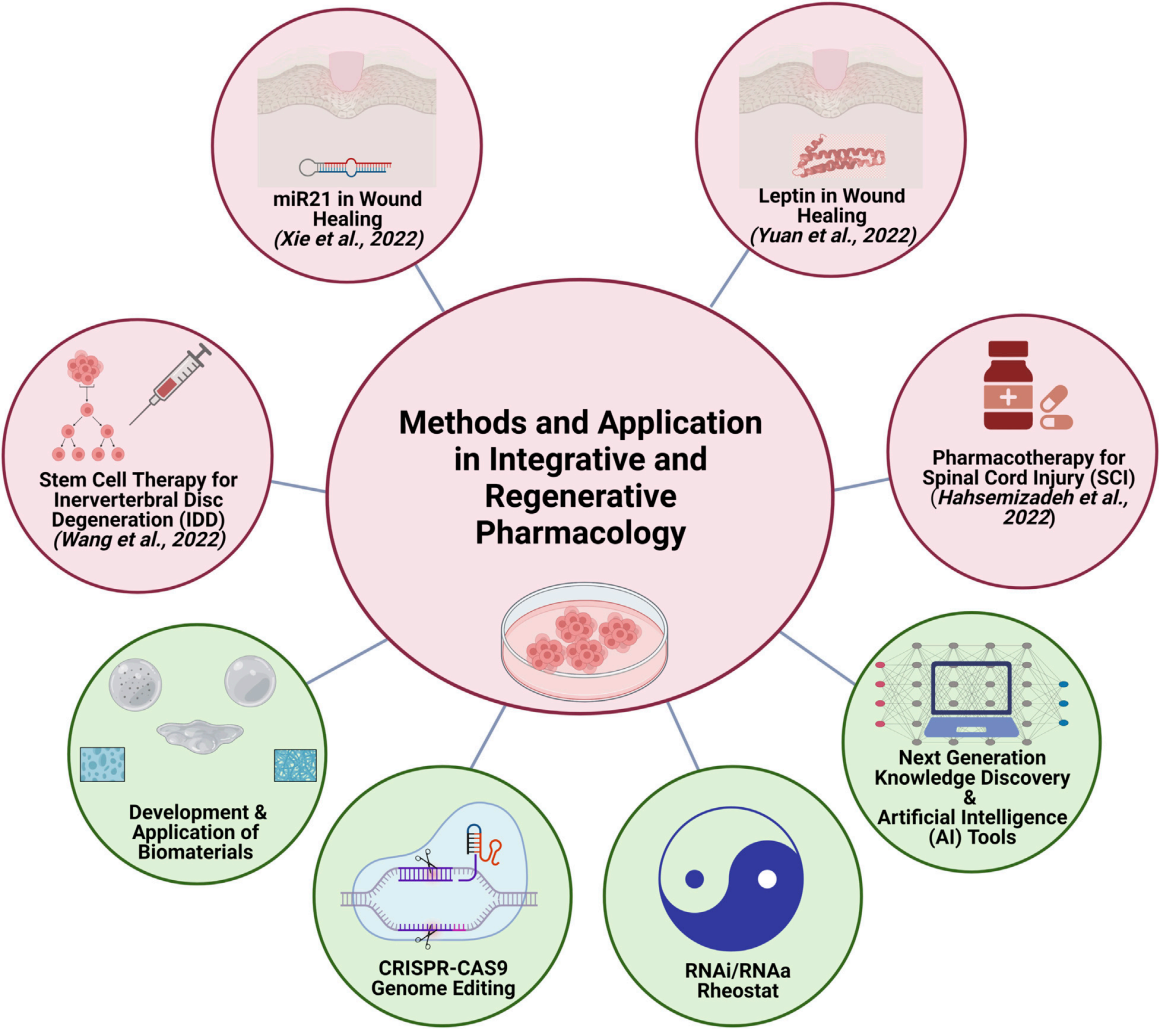
Methods and Application in Integrative and Regenerative Pharmacology. Articles in the special issue (light red circles) address the role of miR21 and leptin in wound healing, stem cell therapy for intervertebral disc degeneration (IDD), and pharmacotherapy for experimental spinal cord injury (SCI) in rats. The future of integrative and regenerative pharmacology (light green circles) depends on research advances in the development and application of biomaterials, CRISPR-CAS9 Genome Editing, RNAi/RNAa rheostat, and next generation knowledge discovery methods and artificial intelligence (AI) tools (*Illustration created using BioRender*).

Advances in the field of biomaterials for stem cell and drug delivery in the last decade have given a much-needed boost to the development of regenerative medicine (Sankar et al., 2017; Farokhi et al., 2021; Pishavar et al., 2021; Irfan et al., 2022; Maruthi and Ramakrishna, 2022). The research progress and development in the field of RNA interference (RNAi) and RNA activation (RNAa) (Li et al., 2006; Pushparaj et al., 2008a; Pushparaj et al., 2008b; Li, 2017; Tan et al., 2021), CRISPR-CAS9 gene editing methods, and the potential application of next-generation knowledge discovery platforms in basic and translational research (Pushparaj et al., 2022) in integrative and regenerative pharmacology are essential for the development of personalized therapies (Figure 1).

## Conclusion and future directions

Articles submitted under the Research Topic highlight the importance of miR21 and leptin signaling in wound healing, the use of human umbilical cord mesenchymal stem cells (HUC-MSCs), BM -MSCs, and adipose-derived stem cells in IDD, and the benefits of bumetanide when administered early after SCI in an experimental animal model. In addition to stem cell-based therapy, CRISPR/cas9 genome editing strategies are the most recent advances that have contributed to the advancement of life sciences (Doudna and Charpentier, 2014). In the future, it is important to apply CRISPR/cas9 genome editing in stem cells and test it using *in vitro* and *in vivo* disease models (Ohmori et al., 2017; Carlson-Stevermer and Saha, 2017; Saha et al., 2021; Nakano et al., 2022) to develop personalized therapy. However, advances in the development and application of biomaterials, next-generation knowledge discovery and artificial intelligence (AI) tools, RNAi/RNAa rheostat, novel drugs, and CRISPR-CAS9 genome editing methods will determine the success of translating research findings in integrative and regenerative pharmacology into the clinic.
